# Utilization of the CometChip assay for detecting PAH-induced DNA bulky adducts in a 3D primary human bronchial epithelial cell model

**DOI:** 10.1016/j.tox.2025.154241

**Published:** 2025-07-23

**Authors:** Victoria C. Colvin, Norah A. Owiti, Bevin P. Engelward, Susan C. Tilton

**Affiliations:** a Department of Environmental and Molecular Toxicology, USA; b OSU/PNNL Superfund Research Program, Oregon State University, Corvallis, OR, USA; c Biological Engineering, Massachusetts Institute of Technology, Cambridge, MA, USA

**Keywords:** Polycyclic aromatic hydrocarbons, Benzo[*a*]pyrene, DNA damage, Comet assay, Cytochrome P450 1A1, Bronchial epithelial cells

## Abstract

Polycyclic aromatic hydrocarbons (PAHs), which are formed during incomplete combustion of organic materials, may cause cancer through DNA damage mediated by formation of bulky DNA adducts from PAH reactive metabolites. The airway epithelium is a primary route of exposure for inhaled PAHs, and primary human bronchial epithelial cells (HBECs) in monolayer or organotypic cultures offer a more realistic testing scenario compared to traditional cell lines. However, lack of knowledge about their capacity to mediate DNA damage through generation of reactive chemical intermediates limits their use in quantitative studies for toxicity assessment or predictive modeling compared to *in vivo* studies. In this study, we explored the capacity of monolayer HBECs to generate DNA damage from metabolic activation of benzo[*a*]pyrene (BAP, 0.001 – 1 μg/mL, 24 h) using the high-throughput CometChip assay in comparison to HepG2 and MEF cells, as positive and negative metabolic controls, respectively. The CometChip assay was further adapted to evaluate DNA damage in HBECs cultured at the air-liquid interface (ALI) exposed to BAP (0.04–1.14 μg/cm^2^, 24 h). Monolayer and ALI-HBECs displayed a statistically significant increase in DNA damage from BAP exposure with repair trapping agents in a dose-dependent manner similar to the response from HepG2 cells. Monolayer HBECs also showed a greater sensitivity to DNA damage compared to ALI-HBECs, which correlated with induction of CYP1A1 activity at similar exposure conditions. Results from the CometChip assay were also observed at lower BAP concentrations compared to CYP1A1 activity, cytotoxicity, or barrier integrity disruption demonstrating the sensitivity of the CometChip assay.

## 1. Introduction

Polycyclic aromatic hydrocarbons (PAHs) are a large class of organic chemicals with two or more fused benzene rings (Kim et al., 2013; Lawal, 2017). There are many natural and anthropogenic sources leading to their presence as common environmental air pollutants including wildfires, fossil fuel combustion, tobacco smoking, etc. (Lee et al., 2004; Masclet et al., 1986; Mitra and Ray, 1995; Nikolaou et al., 1984; Yuan et al., 2008; Zhang and Tao, 2009). PAHs have shown potential for mutagenicity, genotoxicity, and carcinogenicity, and 16 PAHs have been selected as priority pollutants by the US EPA including benzo [*a*]pyrene (BAP), which is commonly utilized as a reference for the class of PAHs due to knowledge about its metabolism, toxicity and carcinogenicity (Bosetti et al., 2007; Bukowska et al., 2022; Chang et al., 2019; Hermann, 1981; Li et al., 2001; Miller and Ramos, 2001; Pfeifer et al., 2002; Widziewicz et al., 2017; World Health Organization, 2010). In order to exert toxicity, parent PAHs often require metabolic activation to form reactive intermediates. For example, BAP can be metabolized by xenobiotic metabolizing enzymes such as CYP1A1 and epoxide hydrolase to form benzo[*a*]pyrene-7,8-diol-9,10-epoxide (BPDE) (Alexandrov et al., 2010; Autrup et al., 1980; Autrup et al., 1982; Barhoumi et al., 2014; Boei et al., 2017; Bukowska et al., 2022; Gelboin, 1980; Jernström et al., 1984; Miller and Ramos, 2001; Rojas et al., 1992; Uppstad et al., 2010). BPDE can then react with DNA to form an adduct on the guanine nucleotide (dG-BPDE) (Alexandrov et al., 2010; Autrup et al., 1980; Autrup et al., 1982; Binková et al., 2000; Boysen and Hecht, 2003; Bukowska et al., 2022; Hanelt et al., 1997; Li et al., 2001; Marie et al., 2008; Miller and Ramos, 2001; Rojas et al., 2004; Villalta et al., 2017). dG-BPDE may remain as a bulky adduct or can be removed by nucleotide excision repair (NER). Either pathway may lead to an incorrect base being inserted creating a mutation that could impact cellular processes that drive cancer (Alexandrov et al., 2010; Binková et al., 2000; Bukowska et al., 2022; Deng et al., 2018; Hemminki, 1993; Lagerqvist et al., 2011; Miller and Ramos, 2001; Pal et al., 2022; Pfeifer et al., 2002; Tung et al., 2014).

Humans are most commonly exposed to PAHs by inhalation or ingestion with many PAHs being detectable in most human tissues, and exposure to PAHs can reduce lung function, even for people with no previous conditions (Cakmak et al., 2017; Kim et al., 2013; World Health Organization, 2010). The lung is a major target organ of PAH toxicity from inhalation exposures and is vulnerable to increased carcinogenicity from reactive intermediates (Autrup et al., 1980; Boffetta et al., 1997; Boysen and Hecht, 2003; Deng et al., 2018; Miller and Ramos, 2001; Moorthy et al., 2015; Widziewicz et al., 2017). As research shifts away from animal testing, there is a need for more validated *in vitro* models and *in vitro* genotoxicity assays for PAH toxicity testing. Complex *in vitro* systems, such as 3D or organotypically grown cell cultures, have great potential as a replacement for animal testing by allowing for cellular differentiation and organ-like functionality *in vitro* (Hayden and Harbell, 2021; Shamir and Ewald, 2014). There is a growing focus on respiratory 3D or air-liquid interface (ALI) models that provide greater complexity and similarity to the *in vivo* human lung morphology, functionality, and exposure response (Boei et al., 2017; Cao et al., 2021; Chang et al., 2019; Chang et al., 2020; Dvorak et al., 2011; Fields et al., 2005; Ghio et al., 2013; Hiemstra et al., 2018; Mastalerz et al., 2022; Mathis et al., 2013; Newland et al., 2011; Qin et al., 2019; Rivera et al., 2022; Pezzulo et al., 2011; Wang et al., 2021). These models can provide more human-relevant mechanistic and toxicity data as compared to immortalized cell lines or *in vivo* models. Although primary human lung cells in advanced cell culture models are becoming more popular in research, their capacity for metabolic activation of PAHs is not completely understood. While new approach methodologies (NAMs), such as 3D culture models, are becoming more accepted by regulatory agencies, there is still a need for mechanistic and kinetic information for new and existing NAMs, and there is a need for validation for specific chemical risk assessments and relevance for human endpoints (Bloch et al., 2024; Sewell et al., 2024). This study explores the DNA damage response from BAP exposures in both monolayer and ALI cultured primary human bronchial epithelial cells (HBECs) under conditions requiring metabolic activation into BPDE, leading to dG-BPDE adduct formation and removal.

While there currently exists several methods for observing genotoxicity after exposures, many of these methods were developed for *in vivo* models or immortalized cell lines. Furthermore, some methods present challenges, such as a high false positive rate and low throughput (Corvi and Madia, 2017; Steinberg et al., 2016). Therefore, there is a need to develop higher-throughput and more accurate genotoxicity tests with improved human extrapolation. The comet assay is a gel electrophoresis method wherein DNA harboring single strand breaks can be detected by its ability to migrate through agarose (unrepaired DNA is highly supercoiled and thus is resistant to migration). This is a commonly used method for observing DNA damage after exposures and has proven useful in observing DNA damage from many compounds (Chao and Engelward, 2020; Chan et al., 2016; Collins, 2004; Deng et al., 2018; Gedik et al., 1992; Mutamba et al., 2011; Olive and Banáth, 2006; Qin et al., 2019; Singh et al., 1988). However, the traditional comet assay can be very difficult to run with high time, labor, and fiscal costs for a small data output (Chao and Engelward, 2020; Cordelli et al., 2021; Ngo et al., 2020; Olive and Banáth, 2006; Sykora et al., 2018a). In order to mitigate these limitations, the CometChip assay was developed as a higher-throughput method for DNA damage analysis (Chao and Engelward, 2020; Ge et al., 2014; Ge et al., 2015; Ge et al., 2012; Ngo et al., 2020; Wood et al., 2010). In this assay, single cells are loaded into micron-scale agarose wells in an array that equally spaces cells and positions them in a singular focal plane. This setup allows for greater number of in-focus single cells and automated imaging and analysis. The innovations of the CometChip greatly reduce time, labor, and cost while also increasing toxicity testing throughput and statistical power (Chao and Engelward, 2020; Ngo et al., 2020; Sykora et al., 2018a, 2018b; Wood et al., 2010). The CometChip has been utilized in several *in vivo* and *in vitro* systems for toxicity testing of many compounds including alkylanilines, allergens, engineered nanomaterials, pathogens, peroxides, therapeutics, and PAHs (Barranger and Le Hégarat, 2022; Boyadzhiev et al., 2022; Buick et al., 2022; Chan et al., 2016; Chan et al., 2017; Chao et al., 2012; Fang et al., 2018; Ge et al., 2013; Ge et al., 2015; Ge et al., 2021; Li et al., 2022; Li et al., 2015; Mutamba et al., 2011; Ngo et al., 2021; Ortiz-Sánchez et al., 2022; Owiti et al., 2022; Parrish et al., 2018; Rosenthal et al., 2023; Seo et al., 2019; Seo et al., 2020; Seo et al., 2022; Sonavane et al., 2018; Sotiriou et al., 2014; Sykora et al., 2018a, 2018b; Tay et al., 2020; Wang et al., 2021; Watson et al., 2014; Weingeist et al., 2013).

While the traditional comet assay is sensitive to single strand breaks, it cannot detect unrepaired bulky lesions because their presence does not impact DNA migration. To overcome this limitation, a repair-trapping method was developed. Specifically, during NER of bulky lesions, the DNA is cleaved to enable repair synthesis. As such, by blocking DNA replication, the NER-initiated single strand break can be trapped, which essentially converts an undetectable bulky lesion into a detectable single strand break. Hydroxyurea (HU) and 1-β-D-arabinofuranosyl cytosine (AraC) have been shown previously to be effective for NER repair trapping (Chao and Engelward, 2020; Ngo et al., 2020; Fram and Kufe, 1985). While the CometChip assay and its modifications are being used more broadly, its reliability and reproducibility should be investigated when using the assay with new culture systems and test compounds. In this study, we have validated the CometChip assay as a tool for observing DNA repair intermediates that form as a consequence of adduct removal in HBECs following BAP exposure.

Here, we assessed DNA damage induced by the metabolic activation of BAP in monolayer and organotypically cultured HBECs by utilizing the CometChip assay to observe DNA damage from dG-BPDE adduct removal after BAP exposure. We made comparisons between monolayer and organotypically cultured HBEC capacity for BAP metabolism and adaptability of the CometChip assay for the two culture systems. This study demonstrates the versatility of the CometChip assay as a high-throughput and reproducible genotoxicity assay for both simple and more complex *in vitro* systems. Additionally, it demonstrates the ability of ALI cultured HBECs to generate single strand break repair intermediates from PAHs, thus expanding the utility of this culture system for understanding genotoxicity in humans.

## 2. Methods

### 2.1. Chemicals and reagents

Gibco^™^ high glucose Dulbecco’s Modified Eagle Medium, Cytiva HyClone^™^ FetalClone^™^ II Serum, Cytiva HyClone^™^ penicillin and streptomycin, Gibco^™^ GlutaMAX^™^, trypsin, and SYBR^™^ Gold Nucleic Acid Gel Stain were purchased from Thermo Fisher Scientific, Waltham, MA. PneumaCult^™^-Ex Plus medium, PneumaCult^™^-Ex medium, and PneumaCult^™^-ALI medium were purchased from STEMCELL Technologies, Vancouver, Canada. Ethyl methanesulfonate (EMS, CAS# 62-50-0, 99 %), hydroxyurea (HU, CAS# 127-07-1, 98 %), β-D-arabinofuranoside cytosine (AraC, CAS# 147-94-4, ≥98 %), trypsin inhibitor, and collagenase were purchased from MilliporeSigma, St Louis, MO. Benzo[*a*]pyrene (BAP, CAS# 50-32-8, 96 %) was purchased from Beantown Chemical, Hudson, NH. Tetrachlorodibenzo-p-dioxin (TCDD, CAS# 1746-01-6) was gifted from Dr. Robyn Tanguay.

### 2.2. Cell culture

All cell types were cultured at 37°C and 5 % CO_2_. Complete media (DMEM) was prepared with Gibco^™^ high glucose Dulbecco’s Modified Eagle Medium, 10 % Cytiva HyClone^™^ FetalClone^™^ II Serum, 100 U/mL Cytiva HyClone^™^ penicillin and streptomycin, and 1x Gibco^™^ GlutaMAX^™^. Mouse embryonic fibroblasts (MEF C57BL/6, ATCC, Manassas, VA) were self-immortalized by passaging cells in culture flasks with complete DMEM once a week until cells began to divide and expand. Cryo-preserved immortalized MEFs were cultured up to passage 6. Cells were transferred to 96-well plates with 20,000 cells per well and allowed to adhere and expand for 24 h.

Cryo-preserved HepG2 cells (ATCC, Manassas, VA) were cultured in complete DMEM up to passage 6 with media changes every 2–3 days and passaged once a week. Cells were transferred to 96-well plates with 16,000 – 30,000 cells per well and allowed to adhere and expand for 24 h.

For monolayer cultures, cryo-preserved normal primary human bronchial epithelial cells (HBEC, Lonza, Basel, Switzerland) were expanded to passage 6 using PneumaCult^™^-Ex Plus medium with media changes every 2–3 days. Once confluent, cells were transferred to 96-well plates with 16,000 – 18,000 cells per well and allowed to expand to confluency over 1 – 2 days. Culture media was switched to PneumaCult^™^-Ex medium just before treatment.

For organotypic ALI cultures, cryo-preserved HBECs were expanded to passage 4 using PneumaCult^™^-Ex Plus medium with media changes every 2 – 3 days (Colvin et al., 2024; Valdez et al., 2024). Once confluent, cells were transferred to 24-well plates with 0.33 cm^2^ transwell inserts with 9000 cells per insert. Once confluent on the inserts, cells were cultured at the air-liquid interface (ALI) using PneumaCult^™^-ALI medium in the basal chamber to initiate differentiation. Cells were cultured at the ALI for 25 days with basal media changes every 2–3 days and mucus washes using Dulbecco’s Phosphate Buffered Saline (DPBS) on the apical surface once a week starting at 14 days post air-lifting with differentiation status previously confirmed under these conditions (Chang et al., 2019; Valdez et al., 2024).

### 2.3. Chemical exposures

Chemicals were prepared in dimethyl sulfoxide (DMSO) then diluted in culture media for exposure. MEF and HepG2 cells were treated with 100 μL of BAP (0.5, 1, 3, 7.5, or 15 μg/mL) or EMS (3 or 6 mM) in complete DMEM with 0.5 % DMSO vehicle in the presence or absence of 1 mM HU and 10 μM AraC for 24 h. To assess DNA damage, monolayer HBECs were treated with 100 μL of BAP (0.001, 0.01, 0.1, 0.5, or 1 μg/mL) or EMS (3 mM) in media with 0.5 % DMSO vehicle in the presence or absence of 0.25 mM HU and 2.5 μM AraC for 24 h. Monolayer HBEC trapping agent co-treatment concentrations were matched to ALI-HBEC trapping agent co-treatment concentrations to aid in comparisons between the models. To assess CYP1A1 activity, monolayer HBECs were treated with 100 μL of BAP (0.01, 0.1, 0.5, or 1 μg/mL) or TCDD (0.16 ng/mL) in media with 1 % DMSO vehicle for 24 h. Media was collected and immediately used for cytotoxicity analysis or stored at −80 °C.

Prior to exposure, ALI-HBECs were apically washed with DPBS to remove mucus. To assess the route of exposure, ALI-HBECs were apically treated with 25 μL of EMS (75.76 or 757.58 nmol/cm^2^) in DPBS with 0.5 % DMSO vehicle or basally treated with 500 μL of EMS (0.05 or 0.5 mM) in media with 0.5 % DMSO vehicle for 24 h. The total amount of EMS applied apically or basally was kept constant at either 25 or 250 nmoles to assist in comparisons. To assess DNA damage, ALI-HBECs were apically treated with 25 μL of BAP (0.04, 0.08, 0.23, 0.57, 1.14 μg/cm^2^) or EMS (757.58 nmoles/cm^2^) in DPBS with 0.5 % DMSO vehicle in the presence or absence of 0.25 mM HU and 2.5 μM AraC in the basal media for 24 h. ALI-HBEC trapping agent co-treatment concentrations were experimentally determined as the concentrations of HU and AraC that allowed for observation of BAP-induced DNA damage without increasing DNA damage in control samples above 20–30 % (Figure A.1 A) and did not increase cytotoxicity (Figure A.1B). To assess CYP1A1 activity, ALI-HBECs were apically treated with 25 μL of BAP (0.04, 0.08, 0.23, 0.57, 1.14, 2.27 μg/cm^2^) or TCDD (121.2 μg/cm^2^) in DPBS with 1 % DMSO vehicle for 24 h. To assist with comparisons of BAP and EMS dosing between monolayer and organotypic cultures, Table A.1 represents treatments reported by concentration, total amount, and amount per surface area. When comparing between culture models, total nmols of compound added to each system is utilized.

### 2.4. Cytotoxicity

Lactate dehydrogenase (LDH) leakage was measured in media after treatment for all monolayer cultures using the CyQUANT^™^ LDH Cytotoxicity Assay (Invitrogen, Thermo Fisher Scientific, Waltham, MA) per manufacturer’s protocol. For ALI-HBECs, LDH was measured after apical treatment with BAP or EMS as previously described (Chang et al., 2019, 2020; Colvin et al., 2024). The apical surface was rinsed with DPBS, and the wash was passed through a syringe with a 30 G needle to break apart mucus. Cytotoxicity was then evaluated per manufacturer’s protocol using the apical wash. Cytotoxicity was calculated by subtracting absorbance at 680 nm from absorbance at 490 nm using a Synergy HTX plate reader (BioTek, Winooski, VT).

### 2.5. CYP1A1 activity

CYP1A1 activity was measured in monolayer and ALI-HBECs using the P450-Glo^™^ CYP1A1 Assay System (Promega, Madison, WI). CYP1A1 activity in monolayer HBECs was analyzed and normalized to cell viability per manufacturer’s protocol except Luciferin-CEE was diluted in DPBS instead of media to enhance assay sensitivity. The protocol was adapted to assess CYP1A1 activity in ALI-HBECs. Luciferin-CEE solution was diluted in DPBS. ALI-HBEC basal media was replaced with DPBS, and 50 μL of Luciferin-CEE solution was added to the apical surface. After a 3 hr incubation at 37°C, 25 μL of apical liquid was transferred to a white 96-well plate (Thermo Fisher Scientific, Waltham, MA), and 25 μL of detection reagent was added to every well. Luminescence was recorded on a Synergy HTX plate reader (BioTek, Winooski, VT). For all cultures, background luminescence was subtracted and each sample was normalized to its respective cell viability as measured by CellTiter-Glo^®^.

### 2.6. Cell viability

Cell viability was measured in monolayer and ALI-HBECs using the CellTiter-Glo^®^ (CTG) Luminescent Cell Viability Assay (Promega, Madison, WI) per manufacturer’s protocol. Luminescence was recorded on a Synergy HTX plate reader (BioTek, Winooski, VT). Background corrected data were used to normalize CYP1A1 activity analyzed by P450-Glo^™^.

### 2.7. Transepithelial barrier integrity (TEER)

TEER was measured on ALI-HBECs using an epithelial volt-ohmmeter (World Precision Instruments, Sarasota, FL). The volt-ohmmeter was calibrated using a test electrode prior to all measurements. At time zero prior to treatment and at time of collection prior to trypsinization DPBS was added to both apical and basal chambers and resistance was measured (ohms) for each insert. Results were adjusted for background resistance by subtracting resistance measured from an insert without cells.

### 2.8. CometChip formation, cell loading, and imaging

CometChip formation and cell loading were completed as described by Ngo et al. (2020). To obtain a single cell solution for MEF, HepG2, and monolayer HBECs, cells were incubated with 75 μL of 0.025 % trypsin in DPBS at 37°C for 10 min. Collagenase (0.75 mg/mL) was added to the trypsin solution to assist HepG2 and monolayer HBEC dislodging. Trypsinization was paused by adding 75 μL of 1 mg/mL trypsin inhibitor. Cells were scraped from wells and vigorously pipetted to ensure a single cell suspension, and 75 μL of the single cell suspension was loaded into 2 chip macro-wells per sample. To obtain a single cell solution of ALI-HBECs, cells were scraped from the insert with a stainless steel spatula then incubated with 100 μL of 0.025 % trypsin in DPBS substituted with 0.75 mg/mL collagenase at 37°C for 10 min. Trypsinization was paused by adding 100 μL of 1 mg/mL trypsin inhibitor, and cells were vigorously pipetted to ensure a single cell suspension. The cell suspension was further diluted with 400 μL DPBS to prevent cell agglomeration prior to loading into six macro-wells. Cells were incubated in the CometChip for 20 min to allow for cell loading into microwells, excess cells were removed by sheer force, and cells in microwells were trapped by adding a layer of low melting point agarose, as previously described (Ngo et al., 2020).

DNA was stained by submerging the chip in 1x SYBR gold fluorescent stain diluted in DI water protected from light for 20 min. The chip was rinsed with phosphate buffered saline (PBS) and placed in a 96-well plate lid for imaging. Chips were imaged by a Keyence BZ-X710 Fluorescence Microscope (Keyence, Itasca, IL) using a Nikon Plan Fluor 10x/0.30∞/1.2 WD 15.2 objective lens (Keyence, Itasca, IL) taking 81 images per macro-well and using Image Merge BZ-XAnalyzer to stitch images into one image per macro-well. Exposure was set to 1/15 s and black balance was set to remove background fluorescence to improve image analysis in downstream software. CometChip images were loaded into the Trevigen^®^ Comet Analysis Software (bio-techne^®^ R&D Systems, Minneapolis, MN) and scanned to identify useable comets. Identified comets were quality checked for accuracy in head and tail sectioning and image quality. The median percent DNA in the comet tail per sample was used for further analysis.

Monolayer HBECs loaded well into microwells 40 μm in diameter and 50 μm in depth with 50–300 comets analyzed per sample. In order to simplify comparisons between monolayer and organotypic HBECs, DNA repair trapping agent concentrations were matched between the two culture systems at 0.25 mM HU and 2.5 μM AraC. Preliminary studies showed no increase in DNA damage in monolayer HBEC controls at reported trapping agent concentrations (1 mM HU and 10 μM AraC) in contrast to ALI-HBEC controls.

### 2.9. Statistical analysis

For DNA damage and cytotoxicity endpoints, MEF, HepG2, and monolayer HBECs had 6 replicates for vehicle controls and 4 replicates for all other treatments, and data is presented as the average of three independent experiments. For cell viability and CYP1A1 activity endpoints, monolayer HBECs had 6 replicates for each treatment. ALI-HBECs had 3–4 replicates for each treatment for all endpoints. For all endpoints except DNA Damage analysis, results were transformed to vehicle by dividing by respective controls. Statistical analyses for all assays were conducted in GraphPad Prism 10.4.1 (Dotmatics, Boston, MA). Significance of treatments was determined by a one-way ANOVA using Dunnett’s post-hoc test for BAP treatments or *t*-test for positive control treatments. A significance level cutoff was defined at an adjusted p-value of 0.05.

## 3. Results

The primary goal of the current study is to quantify BAP-induced DNA damage in 2D and 3D *in vitro* lung model systems using the repair-trapping CometChip approach that enables detection of bulky lesions by the presence of downstream NER single strand break intermediates. Primary HBECs were cultured in a monolayer format and compared to HepG2 and MEFs, which serve as positive and negative controls for BAP metabolism, respectively, to evaluate the metabolic competency of monolayer HBECs. The CometChip assay was then adapted for use with ALI-HBECs by adjusting the DNA repair trapping agent concentrations to observe single strand breaks that form after BAP exposure as a consequence of DNA adduct removal by NER. CometChip results were evaluated in context of endpoints for cytotoxicity, cell viability, CYP1A1 activity, and barrier integrity after BAP exposure to assess CometChip sensitivity as compared to other toxicity endpoints. Finally, CometChip results were evaluated for sensitivity and reproducibility by analyzing results from three independent experiments in monolayer HBECs and two independent experiments in ALI-HBECs.

### 3.1. BAP toxicity in monolayer cultures

**Fig. 1.**
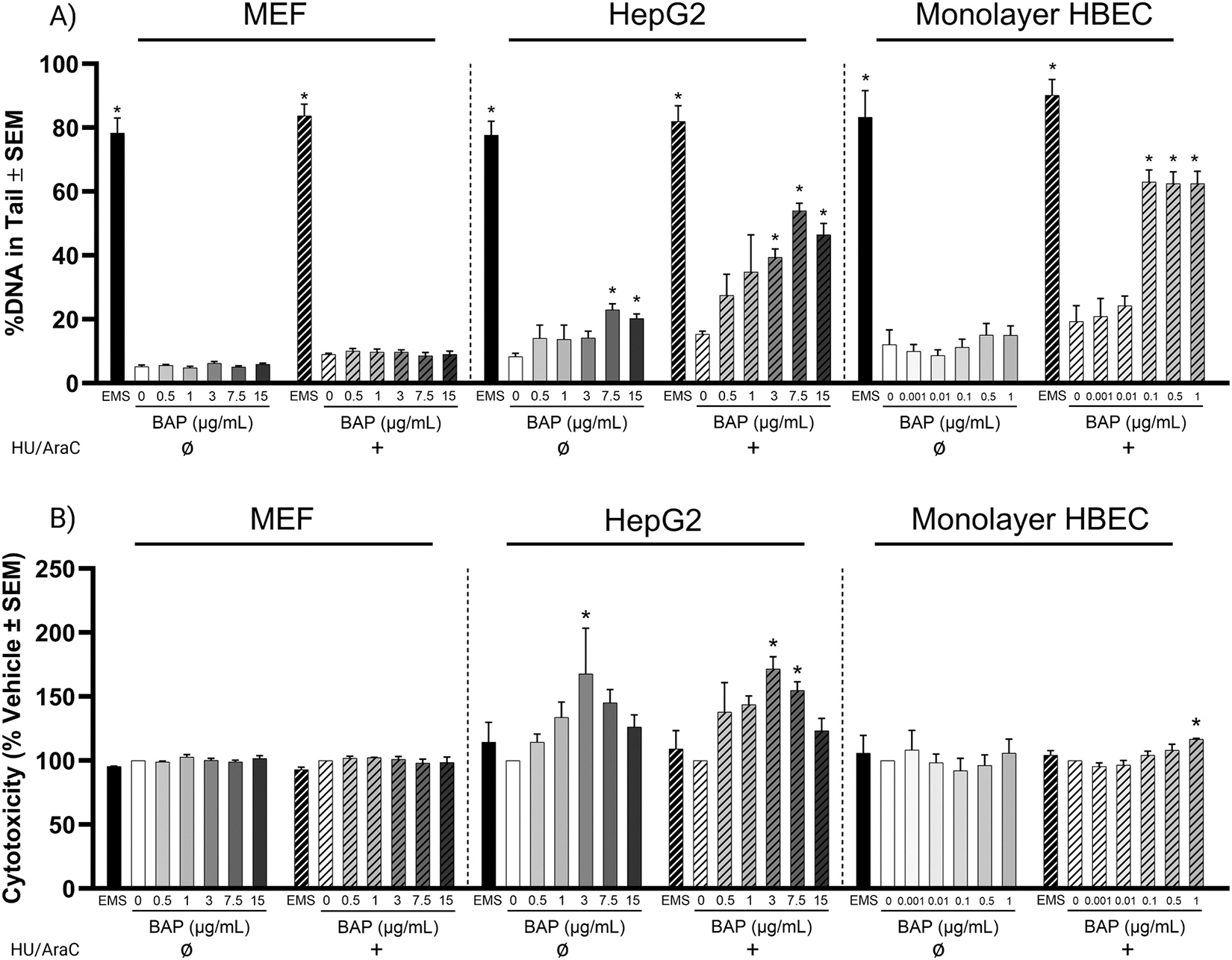
DNA damage and cytotoxicity in MEF, HepG2, and monolayer HBECs with and without DNA repair trapping agent co-treatment as measured by CometChip and LDH leakage, respectively. A) DNA damage represented by the median % DNA in the comet tail averaged over 3 identical experiments. B) Cytotoxicity as represented by the average % change normalized to the vehicle control of 3 identical experiments. Solid bars represent samples without trapping agent co-treatment. Hashed bars represent samples co-treated with DNA repair trapping agents. Error bars represent the standard error of the means. Significance was evaluated using a one-way ANOVA with Dunnett’s post-hoc test compared to the vehicle control for BAP treatments or *t*-test compared to the vehicle control for EMS treatment (* p_adj_ < 0.05).

To evaluate the ability of HBECs to generate NER-induced single strand breaks from metabolically activated PAHs, percent tail DNA was measured after BAP exposure in monolayer HBECs compared to two cell lines, HEPG2 and MEF, with and without metabolic capabilities, respectively (Fig. 1A). EMS treatment, a direct DNA damaging agent, served as a positive DNA damage control and induced significant DNA damage in all cultures regardless of trapping agent co-treatment. As expected, BAP treatment did not induce DNA damage in MEF cultures, which do not express P450s that metabolize BAP. BAP treatment induced DNA damage in HepG2 cultures, a positive BAP metabolism control culture, in a dose-dependent manner which was enhanced by trapping agent co-treatment showing a significant response at and above 3 μg/mL of BAP. Vehicle exposure (in the presence and absence of trapping agents) and BAP exposure without trapping agent co-treatment induced ~10–20 % DNA damage in monolayer HBECs, which was consistent with other cell types. BAP treatment induced DNA damage in monolayer HBEC cultures with trapping agent co-treatment showing a significant response at and above 0.1 μg/mL of BAP. DNA damage response from BAP treatment in monolayer HBECs was always observed at a maximum response level of ~60 % for all significant concentrations of BAP.

To evaluate cytotoxicity in response to chemical treatments, LDH leakage was measured in the media of all cultures 24 h after treatment (Fig. 1B). EMS and BAP treatment did not induce significant cytotoxicity in MEF cultures at any concentration tested regardless of trapping agent co-treatment. There is a general increase in cytotoxicity when HepG2 are exposed to BAP, which shows significance at 3 μg/mL in the absence of HU/AraCa. Similarly, there is increased toxicity associated with BAP in HepG2 cells co-treated with HU/AraC, which rises to significance at the 3 and 7.5 μg/mL doses. Significance was not observed at 15 μg/mL and may indicate lower solubility at the highest concentration. Monolayer HBEC cultures had significant cytotoxicity only for BAP exposure at 1 μg/mL with trapping agent co-treatment at less than 20 % response, which was not biologically relevant, demonstrating that observed DNA damage results are not due to cytotoxicity.

**Fig. 2.**
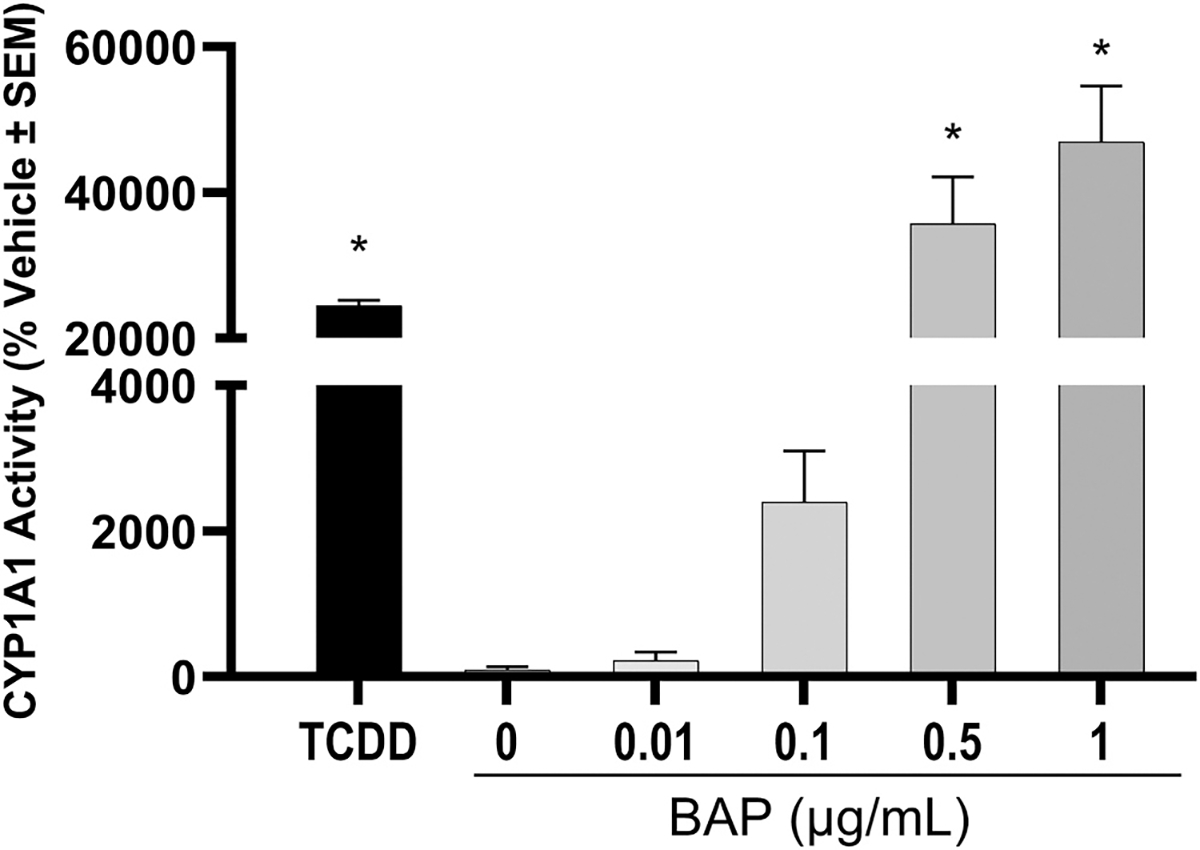
CYP1A1 activity in monolayer HBECs. Data bars represent CYP1A1 activity normalized to number of viable cells and represented by the average % change compared to the vehicle control. Error bars represent the standard error of the means. Significance was evaluated using a one-way ANOVA with Dunnett’s post-hoc test compared to the vehicle control for BAP treatments or *t*-test compared to the vehicle control for TCDD treatment (* padj < 0.05).

To further evaluate monolayer HBEC competency for BAP metabolism and potential for BAP metabolic activation, monolayer HBECs exposed to BAP were evaluated for CYP1A1 activity induction (Fig. 2). BAP exposure significantly induced CYP1A1 activity at and above 0.5 μg/mL which correlates to BAP exposures where significant DNA damage is observed.

### 3.2. Effects of exposure route on DNA damage in ALI-HBECs

**Fig. 3.**
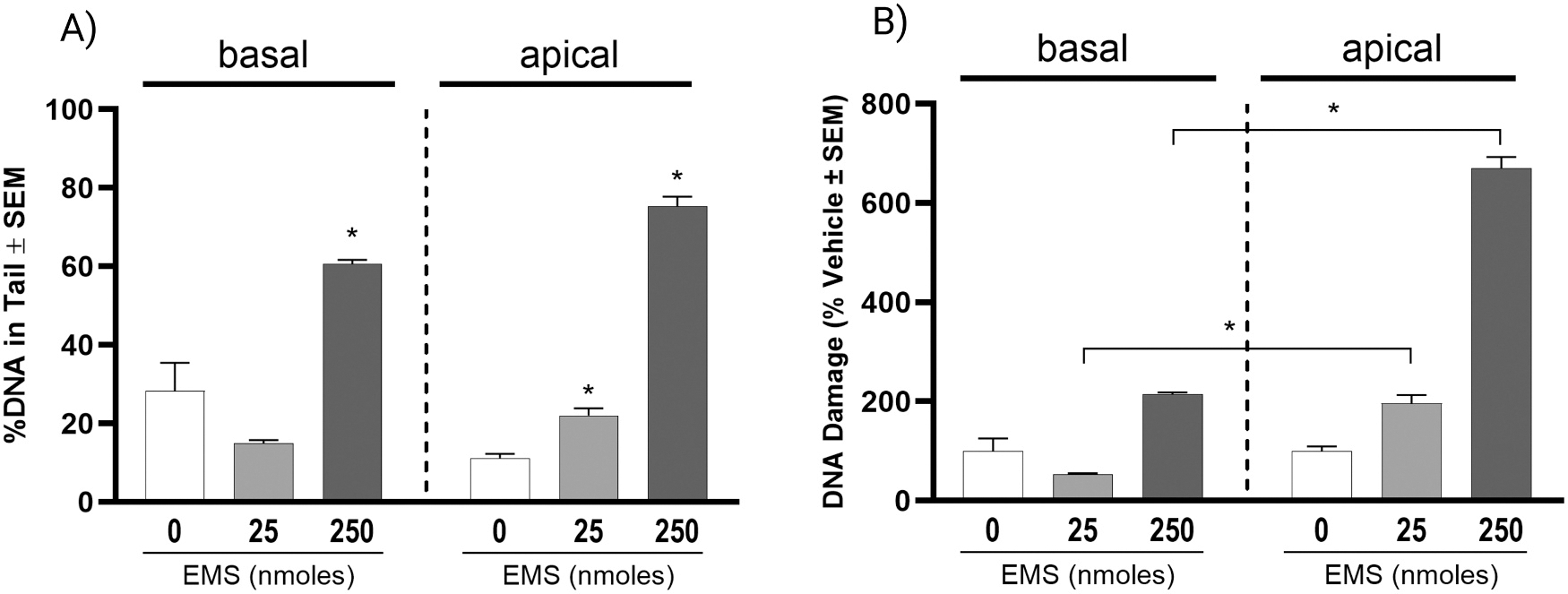
Route of exposure analysis for DNA damage response as measured by CometChip for ALI-HBECs. A) Data bars represent the average of the median % DNA in the comet tail. Significance was evaluated using a one-way ANOVA with Dunnett’s post-hoc test compared to the vehicle control (* p_adj_ < 0.05). B) Data bars represent the average % change normalized to the vehicle control. Significance was evaluated using a *t*-test comparing similar EMS exposures (* p_adj_ < 0.05). Error bars represent the standard error of the means.

In order to evaluate the adaptability of the CometChip assay for more complex *in vitro* systems, we applied the CometChip assay with some modifications to organotypically cultured HBECs. ALI-HBECs can be exposed to compounds through two main routes, the air-exposed apical surface or the media-exposed basal surface. To evaluate potential differences in DNA damage due to these different exposure routes, ALI-HBECs were apically or basally treated with EMS. Basal exposure induced significant DNA damage at 0.5 mM EMS, and apical exposure induced significant DNA damage at 75.76 and 757.58 nmol/cm^2^ EMS (Fig. 3A). There was no significant difference between apical and basal vehicle exposures. When normalized to their respective vehicle controls, there was a significant difference observed between apical and basal exposures at similar EMS doses (Fig. 3B; Table A.1.). EMS did not induce cytotoxicity (Fig. A.2A) or decrease barrier integrity (Fig. A.2B) for any exposure route or treatment of EMS, indicating that observed DNA damage is not due to cytotoxicity. It is also important to note that comparisons were made based on nominal amounts of EMS (total nmol) applied to the basal or apical side. While quantitative dosimetry is unknown, the results demonstrate how either exposure route may be used for observing DNA damage with the CometChip assay in ALI-HBECs. The apical exposure route was selected based on interest in an inhalation exposure scenario as well as potential for greater sensitivity.

### 3.3. BAP toxicity in ALI-HBECs

**Fig. 4.**
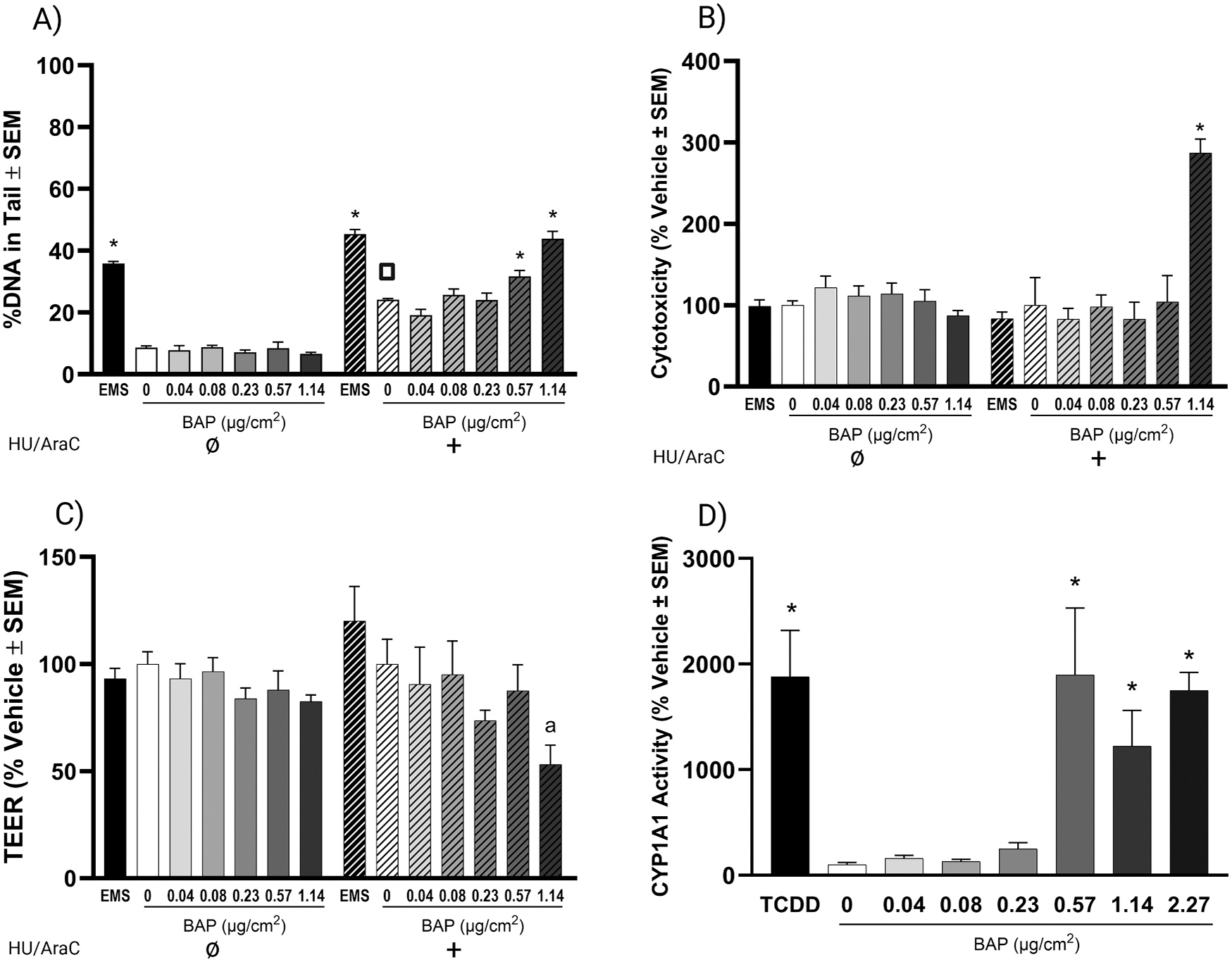
DNA damage, cytotoxicity, barrier integrity, and CYP1A1 activity in ALI-HBECs with and without DNA repair trapping agent co-treatment as measured by CometChip, LDH leakage, TEER, and P450-Glo, respectively. A) DNA damage represented by the average median % DNA in the comet tail. B) Cytotoxicity as represented by the average % change normalized to the vehicle control. C) Barrier integrity as represented by the average % change normalized to the vehicle control. D) CYP1A1 activity normalized to number of viable cells and represented by the average % change compared to the vehicle control. Solid bars represent samples without trapping agent co-treatment. Hashed bars represent samples co-treated with DNA repair trapping agents. Error bars represent the standard error of the means. Significance was evaluated using a one-way ANOVA with Dunnett’s post-hoc test compared to the vehicle control for all BAP treatments or *t*-test compared to the vehicle control for TCDD and EMS treatments (* p_adj_ < 0.05, ^a^ p_adj_ = 0.0597). Square indicates significance evaluated by *t*-test compared to non-trapping agent vehicle control (p_adj_ < 0.05).

ALI-HBECs were evaluated for DNA damage after metabolic activation of BAP (Fig. 4A) and results were validated with a repeat experiment (Fig. A.3). ALI-HBECs loaded well into microwells 40 μm in diameter and 50 μm in depth with 200 – 1400 comets analyzed per sample. EMS induced significant DNA damage regardless of trapping agent co-treatment. For ALI-HBECs, vehicle exposure (in the presence and absence of trapping agents) and BAP exposure without trapping agent co-treatment induced ~10–20 % DNA damage in ALI-HBECs consistent with monolayer HBECs. Depending on the experiment, the level of DNA damage in control treatments in the presence of trapping agents was elevated compared to controls without trapping agents. BAP treatment induced DNA damage in ALI-HBECs with trapping agent co-treatment showing a significant response at and above 0.57 μg/cm^2^ of BAP (Fig. 4A). EMS and BAP induced similar results in a repeat experiment (Figure A.3).

To evaluate cytotoxicity in response to chemical treatments, LDH leakage (Fig. 4B) and barrier integrity (Fig. 4C) were evaluated after 24 h of treatment. ALI-HBECs had significant cytotoxicity only for 1.14 μg/cm^2^ of BAP with trapping agent co-treatment demonstrating that DNA damage results are not due to cytotoxicity. This was matched to a trend (p_adj_ = 0.0597) of decreased barrier integrity for 1.14 μg/cm^2^ of BAP with trapping agent co-treatment.

To evaluate the potential for BAP metabolic activation, ALI-HBECs were evaluated for CYP1A1 activity induction after BAP exposure (Fig. 4D). BAP significantly induced CYP1A1 activity at and above 0.57 μg/cm^2^ similar to the BAP exposure levels that resulted in significant DNA damage.

## 4. Discussion

Advancements in NAMs are rapidly improving our ability to predict genotoxic consequences of exposures. However, their utility for use in chemical risk assessment requires validation. As a part of this validation, NAMs need to be evaluated for their relevance, accuracy, and reproducibility for the cell types being used as well as the types of compounds to be studied. This is particularly important in the context of PAHs, since many PAHs require metabolic activation. Of particular interest is the possibility that more realistic culture conditions enable more robust metabolism. For example, Qin et al. (2019) found that monolayer HBECs are unable to metabolize a nitrosamine and thus are resistant to toxicity, whereas HBECs cultured at the air-liquid interface are susceptible. This study demonstrates an important limitation in studying monolayer HBECs for nitrosamine toxicity assessments. In addition to differences in metabolism associated with differences in cell culture conditions, there are also limitations caused by the use of immortalized cell lines. For example, using the CometChip, Seo et al. (2020) found that primary human hepatocytes are more sensitive to genotoxic agents than immortalized cell lines. Given these prior studies, we set out to test the efficacy of the CometChip approach for studies of PAH-induced DNA damage in HBECs under different culture conditions. We show that the CometChip is indeed effective for detecting BAP-induced DNA damage, and furthermore, that cell culture conditions alter the susceptibility of HBECs to BAP-induced DNA damage.

### 4.1. Metabolic competency of monolayer cultures

In order to assess monolayer HBEC competency for BAP metabolism, monolayer HBEC cells were compared to MEFs and HepG2 cells for their sensitivity to BAP-induced DNA damage. MEFs were used as a negative control cell line because they are metabolically incompetent due to their lack of CYP1A1 expression, a key enzyme in the metabolism of BAP into the genotoxic BPDE metabolite (Alexander et al., 1997; Barhoumi et al., 2014; Bukowska et al., 2022; Moorthy et al., 2015; Uppstad et al., 2010). In contrast, HepG2 cultures are an effective positive control cell line because they express CYP1A1 and CYP1A1 expression is inducible, enabling metabolism of BAP (Castell et al., 2006; Ngo et al., 2020; Urani et al., 1998). Here, we confirmed that exposure to BAP significantly induced CYP1A1 activity and significantly induced DNA damage that was detectable using the repair trapping method of the CometChip suggesting that both monolayer HBECs and HepG2 cells were able to metabolize BAP into the genotoxic BPDE metabolite. Monolayer HBECs were also observed to be much more sensitive to BAP metabolite-induced DNA damage than HepG2 cultures, with statistically significant increases observed at much lower concentrations of BAP based on equivalent dosing (Table A.1.). This result could be attributed to immortalized cell lines having reduced metabolic capabilities, as has been shown previously (Castell et al., 2005, 2006; Seo et al., 2020; Willey et al., 1996). Under our experimental conditions, every concentration of BAP resulted in a significant response for monolayer HBECs, all of which were at about 60 % DNA in tail, suggesting a narrow dose-range for toxicity and the potential for response saturation in HBECs compared to HepG2 cells, which showed a dose-response curve. Of note, however, the dose range for HBEC ranged over several orders of magnitude in order to include analysis of lower concentrations, which may have obscured a possible dose response curve. As such, additional studies are needed to identify the threshold concentration that elicits DNA damage.

### 4.2. CometChip adaptability for complex in vitro systems

We evaluated the adaptability of the CometChip assay for ALI-HBECs by first evaluating DNA damage from EMS, a direct-acting genotoxin that does not require metabolic activation. In a previous study, Wang et al. (2021) observed a statistically significant increase in DNA damage in ALI-HBECs after basal EMS exposure using a slightly modified loading procedure for the CometChip assay. We applied similar techniques in the present study to evaluate basal EMS exposure in ALI-HBECs, and we observed a similar statistically significant increase in DNA damage, demonstrating the reproducibility of the CometChip assay between studies. ALI-HBECs and similar lung *in vitro* models are often treated via apical exposure to mimic exposure of compounds in the lung compared to treatments on the basal side representing systemic exposures (BéruBé et al., 2010; Cao et al., 2021). Therefore, we evaluated potential differences in DNA damage for apical versus basal exposures. Apical exposures resulted in DNA damage following exposure to both 75 and 757 nmol/cm^2^ EMS that was significantly higher than the DNA damage induced by basal exposure, when normalized to their respective controls. This suggests that the apical exposure scenario results in a greater sensitivity for observing DNA damage in ALI-HBECs. Although not explicitly explored in the studies presented here, a possible reason for this finding could be that there are differences in transport mechanisms between the apical and basal surfaces of lung epithelial cells cultured at the ALI (Ali Selo et al., 2019; Barilli et al., 2020; Gumbleton et al., 2011; Nickel et al., 2016; Rotoli et al., 2020).

While the CometChip has been demonstrated to work well for ALI-HBECs exposed to direct DNA damaging agents, further adaptations were needed to apply this assay to PAH exposures where DNA damage results from bulky DNA adduct removal. Preliminary studies in ALI-HBECs using published concentrations of trapping agents (1 mM HU and 10 μM AraC) from *in vitro* monolayer studies resulted in increased DNA damage in control cells. Therefore, varying trapping agent concentrations were investigated to identify the optimal concentrations for ALI-HBECs. Our studies identified 0.25 mM HU and 2.5 μM AraC as the optimal concentrations that allowed for DNA damage observations from BAP exposure without inducing excess DNA damage in the vehicle controls. These results show that a range-finding experiment is a key step when using the repair-trapping CometChip assay for new cell types or for cells cultured under different conditions. Of note, DNA damage in control cells from both HBEC monolayer and ALI culture experiments were approximately 20 % in the presence of trapping agents and ranged 10–20 % without trapping agents. Ngo et al. (2020) also observed increased damage in controls with trapping agent exposure noting that this could result from spontaneous DNA repair, and precautions should be taken to reduce excess damage in controls where possible when applying the CometChip assay in toxicity assessments. For example, reducing trapping agent concentrations can reduce damage in controls for sensitive culture models as demonstrated with ALI-HBECs. Additionally, reducing the concentration of vehicles such as DMSO can help reduce damage in controls as demonstrated here with 0.5 % DMSO in CometChip experiments. Other methods for reducing damage in controls include reducing the amount of time cells are at room temperature particularly during the trypsinization and CometChip loading steps. These results demonstrate the adaptability of the CometChip assay for observing DNA damage in more complex *in vitro* systems.

### 4.3. BAP metabolic activation in simple and complex in vitro systems

As a first step toward assessing DNA damage in ALI-HBECs after metabolic activation of BAP, CYP1A1 activity was evaluated after exposure to BAP and DNA damage was evaluated from BAP exposure in the presence or absence of DNA repair trapping agents. We observed BAP exposure significantly induced CYP1A1 activity and significantly induced DNA damage detectable in the presence of the repair trapping agents, suggesting that ALI-HBECs are capable of BAP metabolism into the genotoxic BPDE metabolite. ALI cultured lung cells have been shown to preserve CYP450 expression and activity similar to levels in *in vivo* lung cells including preserving the inducibility of CYP1A1 (Baxter et al., 2015; Cao et al., 2021; Chang et al., 2019, 2020; Newland et al., 2011; Zhang et al., 2018). BAP exposure has also been shown to result in BPDE formation and BPDE-dG adduct formation in human lung epithelial cell cultures and tissues which correlates with an increased chance of lung cancer (Alexandrov et al., 2010; Autrup et al., 1980; Boysen and Hecht, 2003).

Interestingly, monolayer HBECs were more susceptible to CYP1A1 induction and associated DNA damage compared to ALI-HBECs based on equivalent dosing (Table A.1.). This is opposite to what was seen in HepaRG cells, an immortalized liver cell line, where 3D cultures were observed to be more sensitive to genotoxic agents than 2D cultures, but similar to the trend in BEAS-2B cells, an immortalized lung cell line, where undifferentiated cultures were more sensitive to particle exposures than those cultured at the ALI demonstrating that chemical sensitivity between 2D and 3D cultures could be cell type specific (Ghio et al., 2013; Seo et al., 2022). Comparing CometChip images at similar BAP exposures demonstrates this sensitivity difference where ALI-HBEC comets tend to have smaller and more varied tails compared to monolayer HBECs which have larger comet tails with less variance (Figure A.4). This sensitivity difference could be explained by the morphological differences between monolayer and ALI-HBECs. ALI-HBECs have been shown to mimic *in vivo* lung cell-type proportions with 25 – 30 % basal cells, 25 % secretory cells including club and goblet cells, and 40 – 70 % ciliated cells, and monolayer HBECs have been shown to only consist of basal cells (Boers et al., 1998; Boers et al., 1999; Lumsden et al., 1984; Pezzulo et al., 2011; Schamberger et al., 2015). These differences in cell-type distributions could also contribute to differences in metabolizing enzyme activities where some cell types may have greater potential for CYP450 induction than others. While ALI-HBECs have been shown to express a greater number of CYP450s, monolayer HBECs have been shown to have a greater CYP1A1 inducibility (Cao et al., 2021; Qin et al., 2019). The knowledge around metabolizing enzyme expression and inducibility in the different lung cell types is currently lacking, and more research is needed in this area to fully understand how differences in cell-type specific CYP450 activity may affect the toxicity of compounds reliant on metabolic activation.

No significant DNA damage was detected in monolayer or organotypic HBECs exposed to BAP without trapping agent co-treatment which contrasts results found in monolayer HepG2 and 3D HepaRG cells, another commonly used organotypic culture. HepG2 and HepaRG cultures have shown increased DNA damage from PAH exposure without the use of trapping agents in several previous studies (Barranger and Le Hégarat, 2022; Seo et al., 2019, 2020, 2022). However, Ngo et al. (2020) demonstrated an increased sensitivity in the results when trapping agents were utilized consistent with the results shown here.

The difference in response between monolayer and ALI-HBECs to DNA damage from BAP exposure as observed by the CometChip assay reveals advantages and disadvantages for the two culture models in toxicity assessments. For example, due to their increased sensitivity and shorter culture time, monolayer HBECs may be better suited for high throughput toxicity testing for chemical prioritization while ALI-HBECs may be better suited for mechanistic studies to observe tissue-related responses or differences between lung cell types. Overall, these results demonstrate the applicability and usefulness of the CometChip assay for observing DNA damage from PAH exposures in multiple culture systems and provide evidence for the metabolic competency of monolayer and ALI-HBECs for BAP metabolism.

### 4.4. CometChip reproducibility and sensitivity

In order to validate the CometChip assay as a useful tool for toxicity assessments, the reproducibility of results must be evaluated. Here we present data from a variety of culture systems and exposure methods that demonstrate the reproducibility of the CometChip assay for genotoxicity assessments. The CometChip data from monolayer HBECs, HepG2s, and MEFs exposed to BAP are presented as the average of three identical experiments and typically present a standard error of < 10 % between experiments. Similarly, CometChip data between two identical experiments with ALI-HBECs exposed to BAP resulted in a standard error of < 5 % between experiments. Previous studies also tend to present CometChip data as the average of 2 or more identical experiments when conducted with *in vitro* systems and show similar results with low variability between experiments (Barranger and Le Hégarat, 2022; Buick et al., 2022; Chao et al., 2012; Fang et al., 2018; Ge et al., 2013, 2015; Li et al., 2015; Ortiz-Sánchez et al., 2022; Parrish et al., 2018; Sotiriou et al., 2014; Sykora et al., 2018b; Watson et al., 2014; Weingeist et al., 2013). There has been little previous research on the reproducibility of CometChip results utilizing DNA repair trapping agents for observing DNA damage from single strand breaks. In this study we present data showing that trapping agent co-treatment greatly increases CometChip sensitivity and that results are highly reproducible, indicating that the CometChip assay could aid researchers in toxicity assessments of compounds that are potentially genotoxic.

The current study enables comparison among four endpoints (DNA damage, CYP1A1 activity, cytotoxicity, and barrier function) for both the monolayer and ALI-HBECs. We found that DNA damage, was the most sensitive of the four. Specifically, monolayer HBECs exhibited significant DNA damage from BAP exposure at a concentration 10x lower than for significant cytotoxicity, and ALI-HBECs exhibited significant DNA damage from BAP exposure at an exposure 2x lower than for significant cytotoxicity or loss of barrier integrity. CYP1A1 activity showed similar sensitivity to DNA damage results for ALI-HBECs and slightly lower sensitivity for monolayer HBECs. Similar results have been noted in other studies where significant DNA damage is observed with the CometChip assay well before other endpoints including cytotoxicity or cell viability (Barranger and Le Hégarat, 2022; Seo et al., 2019; Seo et al., 2020; Seo et al., 2022; Wang et al., 2021). This increased sensitivity of the CometChip assay could be a useful tool for researchers during high-throughput screening for the prioritization of potentially genotoxic compounds.

## 5. Conclusions

In summary, the applicability and adaptability of the CometChip assay for observing DNA damage was assessed in conjunction with the evaluation of monolayer and ALI-HBEC capacity for BAP metabolic activation. The CometChip method was adapted for primary human lung cells cultured both as a monolayer and organotypic culture. Results show that the CometChip repair trapping approach is reproducible and sensitive for studies of BAP exposure, and we present several recommendations for adapting the CometChip assay to new culture systems. DNA damage in HBECs was compared to a positive and negative control cell line to evaluate the competency for BAP metabolism, and the CometChip results show both monolayer and ALI-HBECs are capable of creating the carcinogenic BPDE metabolite resulting in DNA damage from dG-BPDE removal. Taken together, this study demonstrates the efficacy of the CometChip in conjunction with repair-trapping agents for detecting bulky lesions. Furthermore, results demonstrate that metabolic activation is critical to this process, and that monolayer HBECs are more sensitive to BAP-induced DNA damage compared to ALI-HBECs. Results demonstrate that the CometChip in conjunction with repair trapping agents is a useful tool in toxicity assessments for potentially genotoxic compounds and demonstrate a novel application of the CometChip in studies of HBECs.

## Supplementary Material

MMC1

Appendix A. Supporting information

Supplementary data associated with this article can be found in the online version at doi:10.1016/j.tox.2025.154241.

## Data Availability

Data will be made available on request.
