# Supplementary material for: Utilization of the CometChip assay for detecting PAH-induced DNA bulky adducts in a 3D primary human bronchial epithelial cell model
Source: Toxicology. Author manuscript; Available in PMC 2025 Aug 15. (PMC12356187; doi:10.1016/j.tox.2025.154241)
Supplement: MMC1 [file NIHMS2100746-supplement-MMC1.docx]

**Appendix A**

**Table A.1.** Unit conversions for HBEC culture treatments.

| **Culture** | **Treatment Compound** | **Exposure** | **Treatment by Concentration** | **Treatment by amount (nmoles)** | **Treatment by Surface Area^1^** |
| --- | --- | --- | --- | --- | --- |
| **Monolayer** | BAP | submerged | 0.001 µg/mL | 0.0004 | NA |
|  |  | submerged | 0.01 µg/mL | 0.004 | NA |
|  |  | submerged | 0.1 µg/mL | 0.04 | NA |
|  |  | submerged | 0.5 µg/mL | 0.20 | NA |
|  |  | submerged | 1 µg/mL | 0.40 | NA |
|  |  | submerged | 15 µg/mL | 1.2 | NA |
|  | TCDD | submerged | 0.16 ng/mL | 4.97e-5 | NA |
|  | EMS | submerged | 3 mM | 300 | NA |
|  |  | submerged | 6 mM | 600 | NA |
| **ALI** | BAP | apical | NA | 0.05 | 0.04 µg/cm^2^ |
|  |  | apical | NA | 0.10 | 0.08 µg/cm^2^ |
|  |  | apical | NA | 0.30 | 0.23 µg/cm^2^ |
|  |  | apical | NA | 0.74 | 0.57 µg/cm^2^ |
|  |  | apical | NA | 1.49 | 1.14 µg/cm^2^ |
|  |  | apical | NA | 2.97 | 2.27 µg/cm^2^ |
|  | TCDD | apical | NA | 0.12e-3 | 121.2 µg/cm^2^ |
|  | EMS | apical | NA | 25 | 75.76 nmol/cm^2^ |
|  |  | apical | NA | 250 | 757.58 nmol/cm^2^ |
|  |  | basal | 0.05 mM | 25 | NA |
|  |  | basal | 0.5 mM | 250 | NA |

^1^0.33 cm^2^ used for surface areas of 96-well plate well and 24-well insert culture area.

**
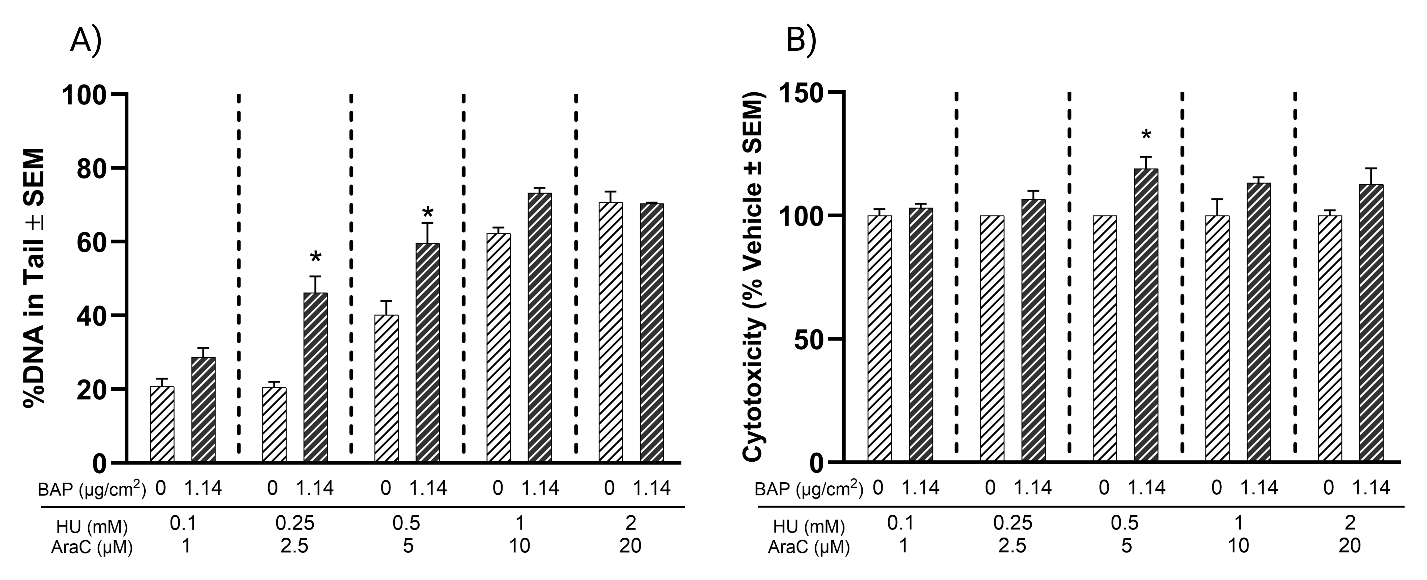
**

**Figure A.1.** Trapping agent concentration analysis for DNA damage response as measured by CometChip for ALI-HBECs. A) DNA damage represented by the average median % DNA in the comet tail. B) Cytotoxicity as represented by the average % change normalized to the vehicle control. Error bars represent the standard error of the means. Significance was evaluated using a t-test compared to the vehicle control (* p_adj_ < 0.05). Samples co-treated with HU (1 or 2 mM) and AraC (10 or 20 µM) had only 2 replicates and were not included in statistical analyses.


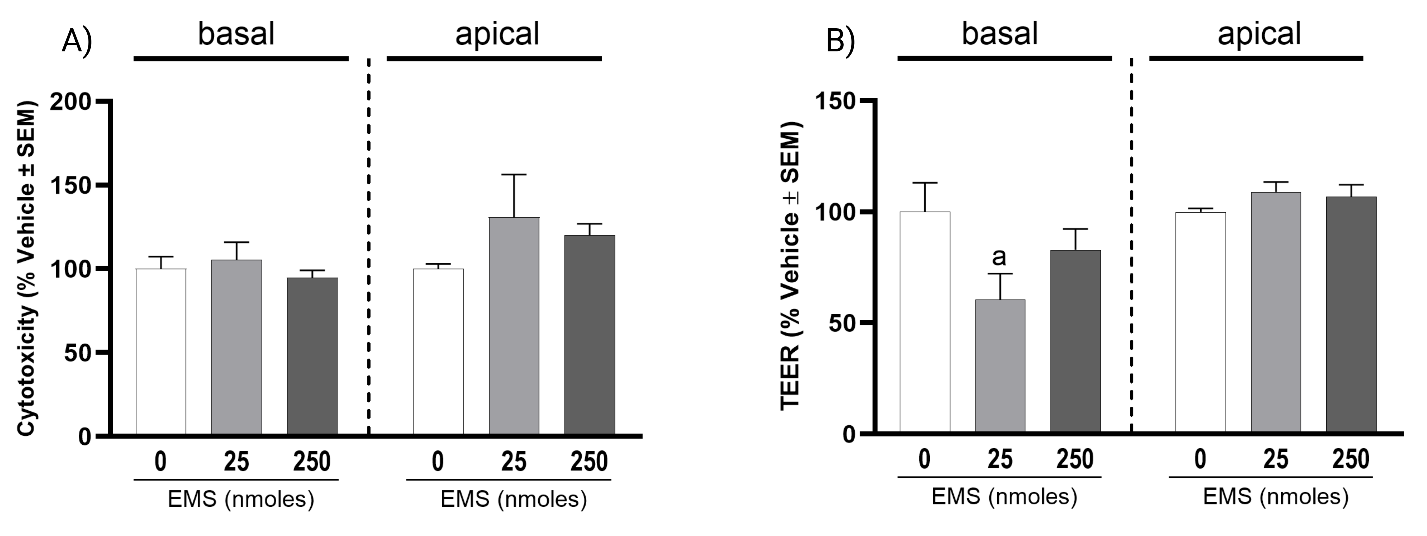


**Figure A.2.** Route of exposure analysis for cytotoxicity and barrier integrity in ALI-HBECs as measured by LDH leakage and TEER, respectively. A) Cytotoxicity as represented by the average % change normalized to the vehicle control. B) Barrier integrity as represented by the average % change normalized to the vehicle control. Error bars represent the standard error of the means. Significance was evaluated using a one-way ANOVA with Dunnett’s post-hoc test compared to the vehicle control (^a^ p_adj_ = 0.0666).


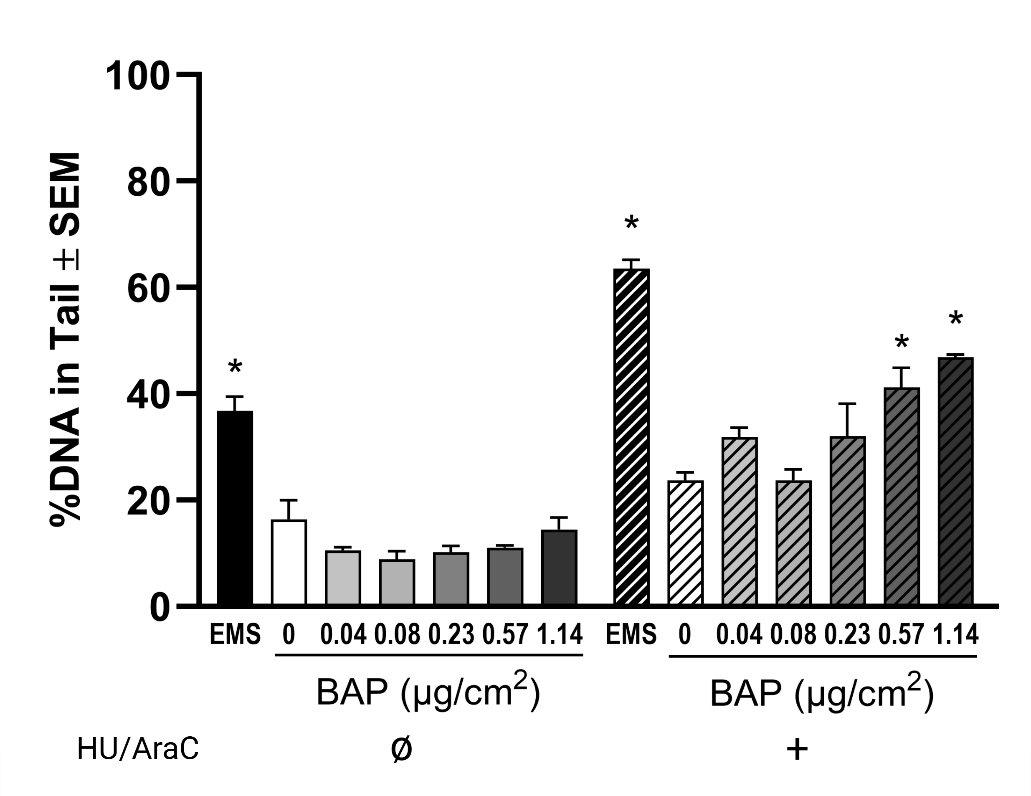


**Figure A.3.** Repeated results of DNA damage in ALI-HBECs with and without DNA repair trapping agent co-treatment as measured by CometChip. DNA damage is represented by the average median % DNA in the comet tail. Hashed bars represent samples co-treated with DNA repair trapping agents. Error bars represent the standard error of the means. Significance was evaluated using a one-way ANOVA with Dunnett’s post-hoc test compared to the vehicle control (* p_adj_ < 0.05).


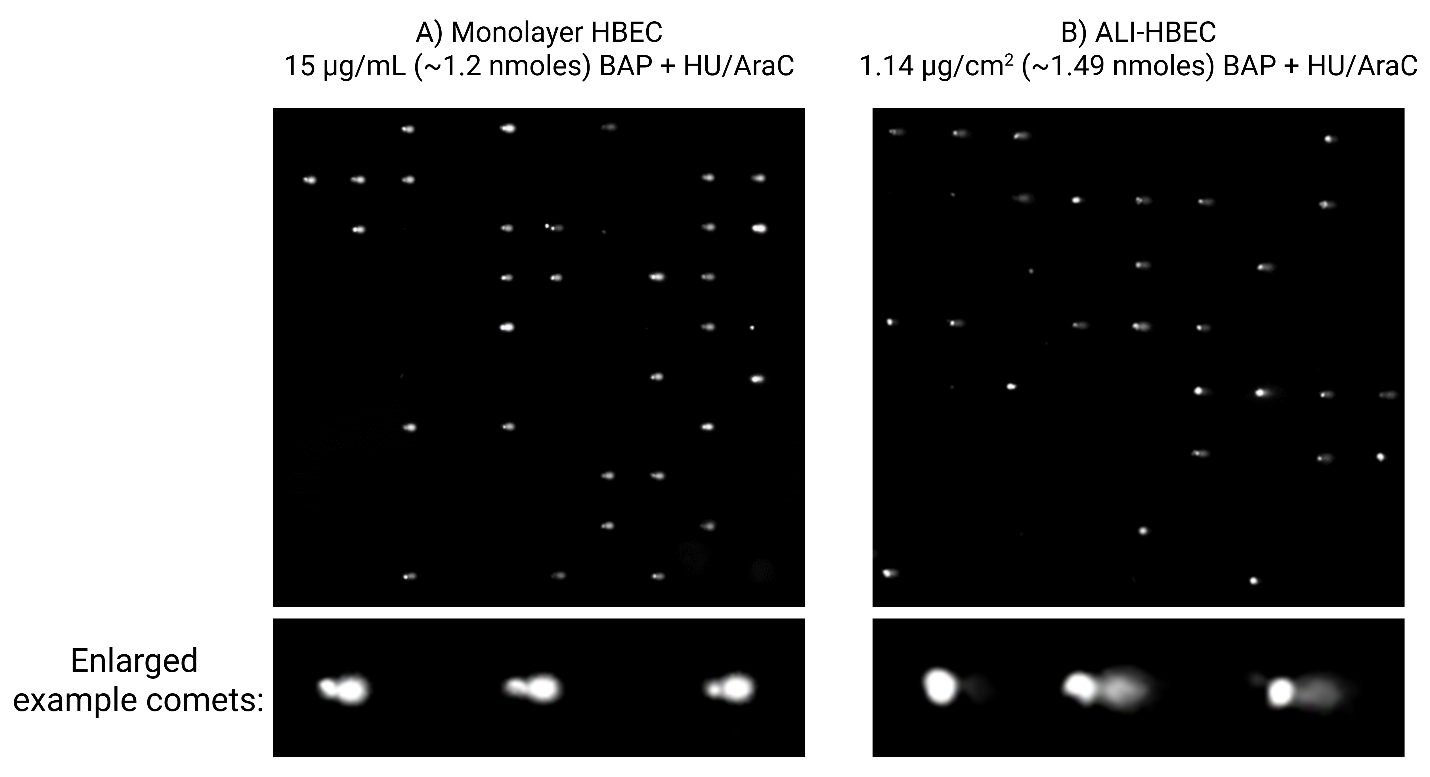


**Figure A.4.** Representative CometChip images for A) monolayer HBECs and B) ALI-HBECs exposed to BAP.
